# The safety of ipsilateral high-frequency repetitive transcranial magnetic stimulation in brain tumour patients

**DOI:** 10.3389/fneur.2026.1814880

**Published:** 2026-06-10

**Authors:** Ahmad M. S. Ali, Priscella Asman, Sai Chilakapati, Michael D. Jenkinson, Rasheed Zakaria, Sujit Prabhu

**Affiliations:** 1 Department of Neurosurgery, The Walton Centre NHS Foundation Trust, Liverpool, United Kingdom; 2 Institute of Systems, Molecular, and Integrative Biology, University of Liverpool, Liverpool, United Kingdom; 3 Department of Neurosurgery, MD Anderson Cancer Center, Houston, TX, United States

**Keywords:** brain tumour, high-frequency stimulation, low-frequency stimulation, post-operative rehabilitation, repetitive transcranial magnetic stimulation

## Abstract

**Background:**

Motor deficits following resection of intrinsic brain tumours impair quality-of-life and survival. Repetitive transcranial magnetic stimulation (rTMS) has shown promise for motor rehabilitation. Only contralesional low-frequency stimulation (CL-LFS) has been tested in this setting. Ipsilesional high-frequency stimulation (IL-HFS), effective in stroke recovery, has not been explored in patients with brain tumours due to several safety concerns, namely: seizure risk, wound healing, and implant heating.

**Methods:**

Four patients received IL-HFS and six received standard CL-LFS following resection of motor-eloquent tumours. Patients were eligible for IL-HFS if ipsilateral motor evoked potentials were preserved. Pre- and post-rTMS myotomal strength (MRC) and functional status (AMPAC 6-Click Daily Activity and Mobility) were compared using the Wilcoxon signed-rank test. Adverse events were recorded.

**Results:**

All patients demonstrated significant improvements in MRC grade (*p* < 0.05). Strength gains were greater in lower-limb myotomes. AMPAC scores improved in both groups. No seizures or heating effects occurred. One IL-HFS patient required wound revision of a third-time operated wound. The same patient had a 30-s syncopal episode during their final rTMS session with no features of seizures. Another patient reported transient (24-h) paraesthesia in the unaffected foot. Finally, another patient with two programmable ventriculoperitoneal shunts *in situ* did not demonstrate any shunt setting changes.

**Conclusion:**

IL-HFS rTMS appears to be safe in the early post-operative period following tumour resection. These preliminary findings support further prospective trials evaluating IL-HFS —alone or combined with CL-LFS— to optimise motor recovery in patients with brain tumours.

## Introduction

Surgical resection of intrinsic brain tumours carries a significant risk of injury to adjacent eloquent cortex (1, 2). When lesions involve the motor cortex or corticospinal tract, the likelihood of post-operative motor deficit is high (2). Such deficits not only impair quality of life but also adversely affect survival by prolonging hospitalisation, delaying rehabilitation, and impeding timely adjuvant chemoradiotherapy (3, 4). Despite advances in pre- and intra-operative techniques to preserve motor function, new deficits remain common (5, 6).

**Figure 1:**
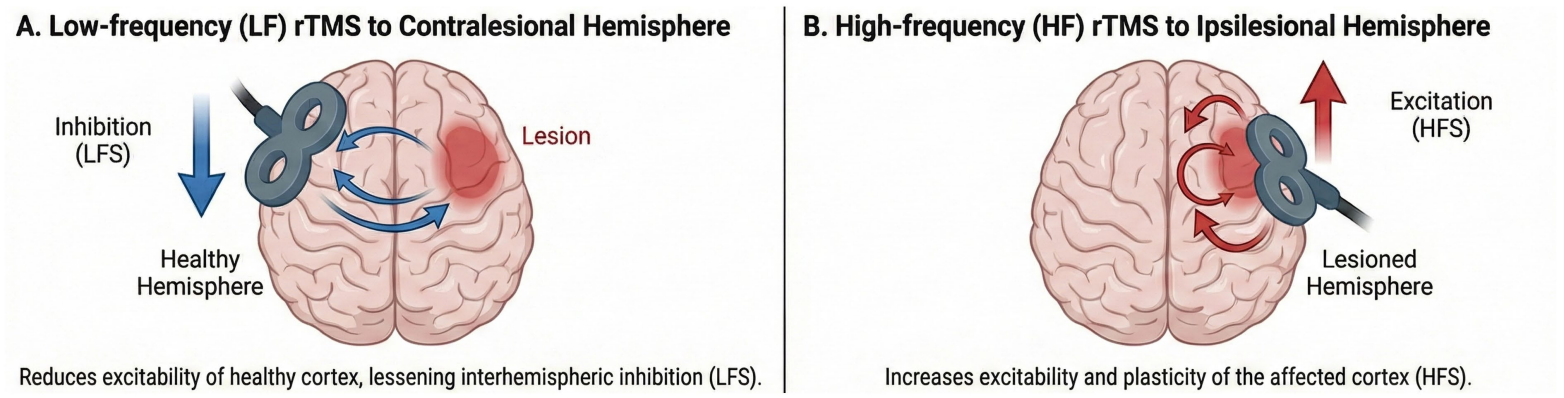
The hypothesised mechanisms through which different frequencies of repetitive transcranial magnetic stimulation (rTMS) may act. **(A)** Low-frequency stimulation to the contralateral hemisphere reduces transcallosal inhibition, allowing improved recovery in the injured hemisphere. **(B)** High-frequency stimulation to the ipsilateral hemisphere directly increases excitability and plasticity. Image generated used Google Gemini.

Repetitive transcranial magnetic stimulation (rTMS) is a non-invasive neuromodulatory technique capable of inducing sustained alterations in cortical excitability (7). High-frequency stimulation (HFS; >5 Hz or intermittent theta-burst) enhances excitability, whereas low-frequency stimulation (LFS; <5 Hz or continuous theta-burst) suppresses it (8). In stroke rehabilitation, LFS applied to the contralesional hemisphere (CL-LFS) is thought to facilitate recovery by attenuating transcallosal inhibition (9, 10), while HFS delivered to the ipsilesional hemisphere (IL-HFS) enhances excitability within peri-lesional motor areas (Figure 1) (11, 12).

In patients with brain tumours, only CL-LFS has been investigated in randomised sham-controlled trials, with mixed outcomes: one study reported benefit over sham stimulation (13), whereas another found only a difference in recovery of hand function but not generalisable to other deficits (14). IL-HFS has not previously been tested in brain tumour patients. There are several reasons for this. IL-HFS carries increased risks and concerns such as stimulation-induced scalp muscle contractions (15) potentially affecting wound healing, elevated seizure risk with high frequency stimulation near surgical resection cavities, and potential heating of cranial fixation metallic implants (16, 17). Given the potential efficacy of IL-HFS in stroke recovery (18) and the mixed evidence regarding CL-LFS for brain tumour rehabilitation, exploration of ipsilesional stimulation after tumour resection is warranted. Here, we report our preliminary experience applying post-operative IL-HFS in four patients with intrinsic brain tumours.

## Methods

A retrospective review of electronic medical records was undertaken to extract clinical and rTMS data from four patients treated with IL-HFS. Equivalent data were obtained for a contemporaneous control group who received the standard CL-LFS protocol. All patients had intrinsic brain tumours involving motor-eloquent regions, defined as proximity to the motor cortex or corticospinal tract. All patients received pre-operative motor mapping using TMS and intra-operative asleep motor mapping using phase reversal to identify the central sulcus and subsequent monopolar stimulation during resection with Motor Evoked Potentials (MEP) monitoring. Each developed a new post-operative motor deficit qualifying them for rTMS and underwent multiple stimulation sessions during their inpatient stay alongside routine physical and occupational therapy. The number and scheduling of rTMS sessions varied owing to working around the patients’ other physical and occupational therapy needs. IL-HFS was applied in four patients, informed by emerging evidence from stroke rehabilitation studies indicating potential efficacy of this paradigm. Eligibility for IL-HFS required preserved ipsilateral motor evoked potentials in the affected limb(s). All patients were closely monitored for adverse events.

Pre- and post-rTMS myotomal strength, documented by a clinician or physiotherapist using the MRC scale, was extracted from medical records. Myotomes assessed included hip flexion, knee flexion and extension, ankle dorsiflexion and plantarflexion, shoulder abduction, elbow flexion and extension, wrist extension, and finger flexion. Functional status was evaluated using the Activity Measure for Post-Acute Care (AMPAC) scales. Specifically, the AMPAC 6-Click Daily Activity and AMPAC 6-Click Mobility scales -both validated ordinal measures of self-reported ability in daily function and mobility respectively- each scored from 6 (completely dependent or immobile) to 24 (completely independent or mobile) (19).

### rTMS protocol

All rTMS sessions were delivered using the Nexstim NBS 5.2.4 system (Nexstim, Helsinki, Finland) equipped with a cooled figure-of-eight coil (outer diameter 70 mm). Post-operative T1-weighted or FLAIR MRI volumes were employed for frameless neuronavigation. Following registration, the resting motor threshold (RMT) was determined using the system’s automated algorithm. MEPs were recorded via surface electrodes placed over the abductor pollicis brevis, first dorsal interosseous, extensor hallucis longus, or tibialis anterior, depending on the target limb. Where ipsilateral MEPs were identifiable, patients received IL-HFS; in their absence, RMT was obtained from the contralateral hemisphere and standard CL-LFS was administered for rehabilitation.

The IL-HFS protocol consisted of 100 pulses delivered at 10 Hz and 100% RMT over 10 s, followed by a 50 s inter-train interval. This sequence was repeated 10 times to deliver 1,000 pulses per cycle, with three such cycles administered per session separated by 30 min intervals, resulting in a total of 3,000 pulses per day. This protocol takes 2 hrs in total. The stimulation was targeted to the hotspot of the affected limb in the ipsilateral motor cortex.

For the CL-LFS protocol, patients received 1,500 pulses delivered at 1 Hz and 100% RMT over 30 s with a 5 s inter-train interval. This sequence was administered once a day and took 30 min in total.

### Statistical analysis

Differences in MRC grades and AMPAC functional scores before and after rTMS were assessed using the Wilcoxon signed-rank test for paired data. For group comparisons of unpaired data, the Mann–Whitney U test was used. Statistical significance was defined as *p* < 0.05. Given the exploratory nature of this pilot study and the small sample size, *p*-values were not corrected for multiple comparisons. All analyses and figure generation were performed in R (version 4.3.1) except for Figure 1 which was generated using Google Gemini.

## Results

### General description

Table 1 summarises the demographic and clinical characteristics of the cohort. Four patients received IL-HFS and six received the standard CL-LFS protocol during the same period. The median age was 64 years, and 44% (*n* = 4) were female. The majority of tumours (67%, *n* = 6) were Glioblastoma, IDH-wildtype, WHO CNS grade 4. There was no difference in the number of TMS sessions between both groups (*p* = 0.564) nor in the number of days over which the TMS course was spread (*p* = 0.108). There was a difference in pre-rTMS lower limb (*p* < 0.001) and upper limb (*p* = 0.002) myotomal strength with worse MRC grades in the CL-LFS group. There was no difference in baseline AMPAC scores (*p* = 0.056) although the trend also indicated a worse functional baseline in the CL-LFS group.

**Table 1:** Summary of patient demographics.

Patient No.	rTMS protocol	Post-op day rTMS started	No. of rTMS sessions	Duration of TMS course	Age at surgery	Gender	Tumour grade/type	Tumour location	ASM	Pre-op weakness	Affected limb
1	IL-HFS	Day20	9	23	64	Male	4	R frontal	No	No	LLL, LUL
2	IL-HFS	Day17	9	19	68	Male	4	R frontal	Yes	No	LLL
3	IL-HFS	Day17	13	19	37	Female	3	L frontal	Yes	No	RLL, RUL
4	IL-HFS	Day87	7	30	60	Male	1	L central	Yes	Yes	RUL
5	CL-LFS	Day8	5	10	56	Male	4	R frontal	Yes	No	LLL, LUL
6	CL-LFS	Day6	8	8	67	Male	4	R cingulate	Yes	No	LLL, LUL
7	CL-LFS	Day9	8	11	68	Female	4	L frontal	Yes	No	RLL, RUL
8	CL-LFS	Day29	9	31	47	Female	Metastatic	L frontal	Yes	No	RLL, RUL
9	CL-LFS	Day8	12	10	71	Female	4	L parietal	Yes	Yes	RLL, RUL
10	CL-LFS	Day14	13	16	47	Male	2	L frontal/ temporal/ insular	Yes	No	RLL, RUL

ASM, Antiseizure medication. UL/LL, Upper limb or Lower limb.

### Motor and functional outcomes

**Figure 2:**
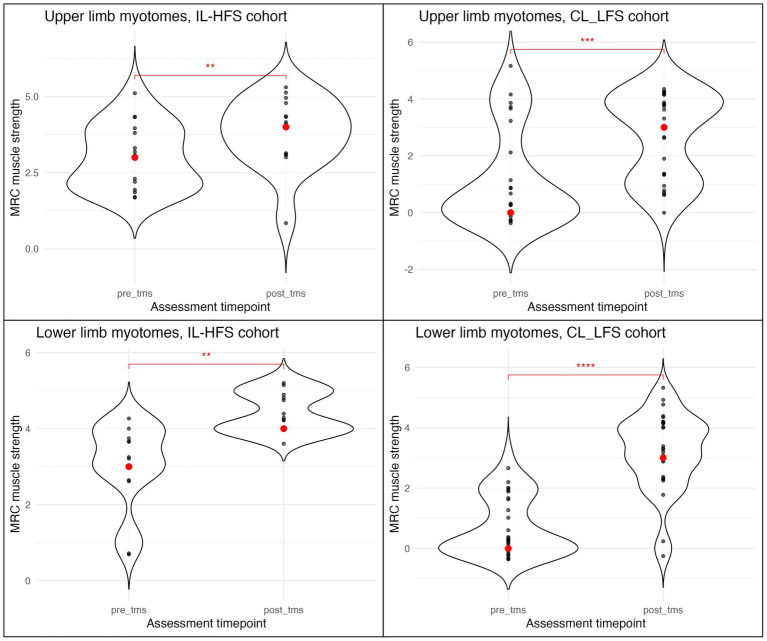
Violin plots demonstrating the Medical Research Council (MRC) grade of muscle strength before and after repetitive transcranial magnetic stimulation (rTMS). The width of each violin plot indicates the distribution density of the data. The red point inside each violin plot indicates the median value. Plots to the right: Contralateral Low-frequency stimulation (CL-LFS) results. Plots to the left: Ipsilateral High-frequency stimulation (IL-HFS) results. Top plots: Upper limb (UL) myotomes. Bottom plots: Lower limb (LL) myotomes. Median MRC for each cohort reported here as [Pre-TMS, Post-TMS] respectively: IL-HFS UL [3, 4], CL-LFS UL [0, 3], IL-HFS LL [3, 4], CL-LFS LL [0, 3].

**Figure 3:**
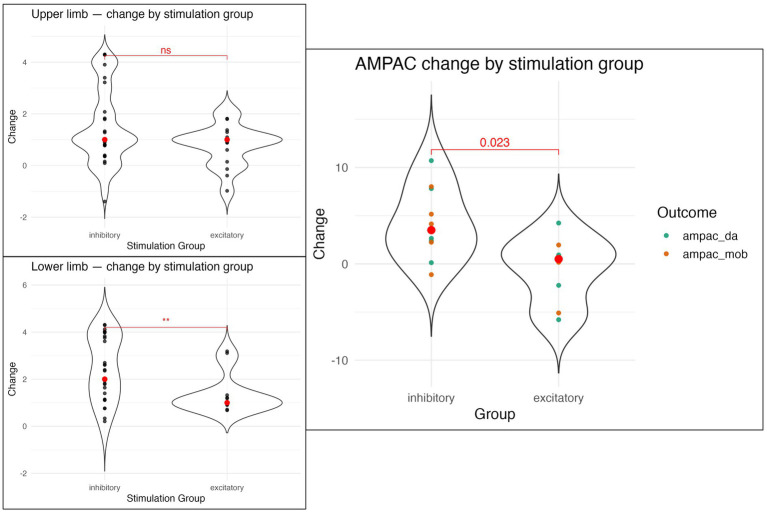
Violin plots comparing the overall *change* in Medical Research Council (MRC) grade and Activity Measure for Post-Acute Care (AMPAC) functional scores between the contralateral low-frequency stimulation (CL-LFS) and ipsilateral high-frequency stimulation (IL-HFS) groups. AMPAC-da is the Daily Activities scale. AMPAC-mob is the Mobility scale. The width of each violin plot indicates the distribution density of the data. The red point inside each violin plot indicates the median value. Median change in scores for each cohort reported here as [Inhibitory, Excitatory] respectively: Upper limb myotomes [+1, +1], Lower limb myotomes [+2, +1], AMPAC-DA [+2.5, −0.5], AMPAC-Mob [+3, +0.5].

Across both stimulation groups, all patients demonstrated statistically significant improvements in MRC grades across upper and lower limb myotomes after rTMS (IL-HFS: lower limb *p* = 0.001, upper limb *p* = 0.009. CL-LFS: lower limb *p* < 0.001, upper limb p < 0.001; Figure 2). In both IL-HFS and CL-LFS cohorts, strength gains were greater in lower- than upper-limb myotomes. At the individual level (Figure 3), the magnitude of change per myotome differed significantly between the two groups only for lower-limb myotomes with greater improvements in the CL-LFS group (*p* = 0.009). There was no difference in the improvements in upper limb myotomes (*p* = 0.209). In parallel, improvements in AMPAC functional scores were also greater in the CL-LFS group (*p* = 0.023).

### Safety profile and adverse events

rTMS was well tolerated across both groups. No headaches, local discomfort, or heating sensations were reported, and no seizures occurred. One IL-HFS patient (Patient 2) reported transient paraesthesia of the unaffected foot, resolving spontaneously within 24 h. Another IL-HFS patient (Patient 3) required post-operative wound revision; however, this individual had undergone two prior operations through the same incision, rendering the wound particularly vulnerable to dehiscence. In the same patient, she had a brief (~30s) syncopal episode during the last of 13 rTMS sessions. She recovered very rapidly from this with no evident post-ictal changes. During this episode no physical movements were seen that could be in keeping with a seizure.

**Figure 4:**
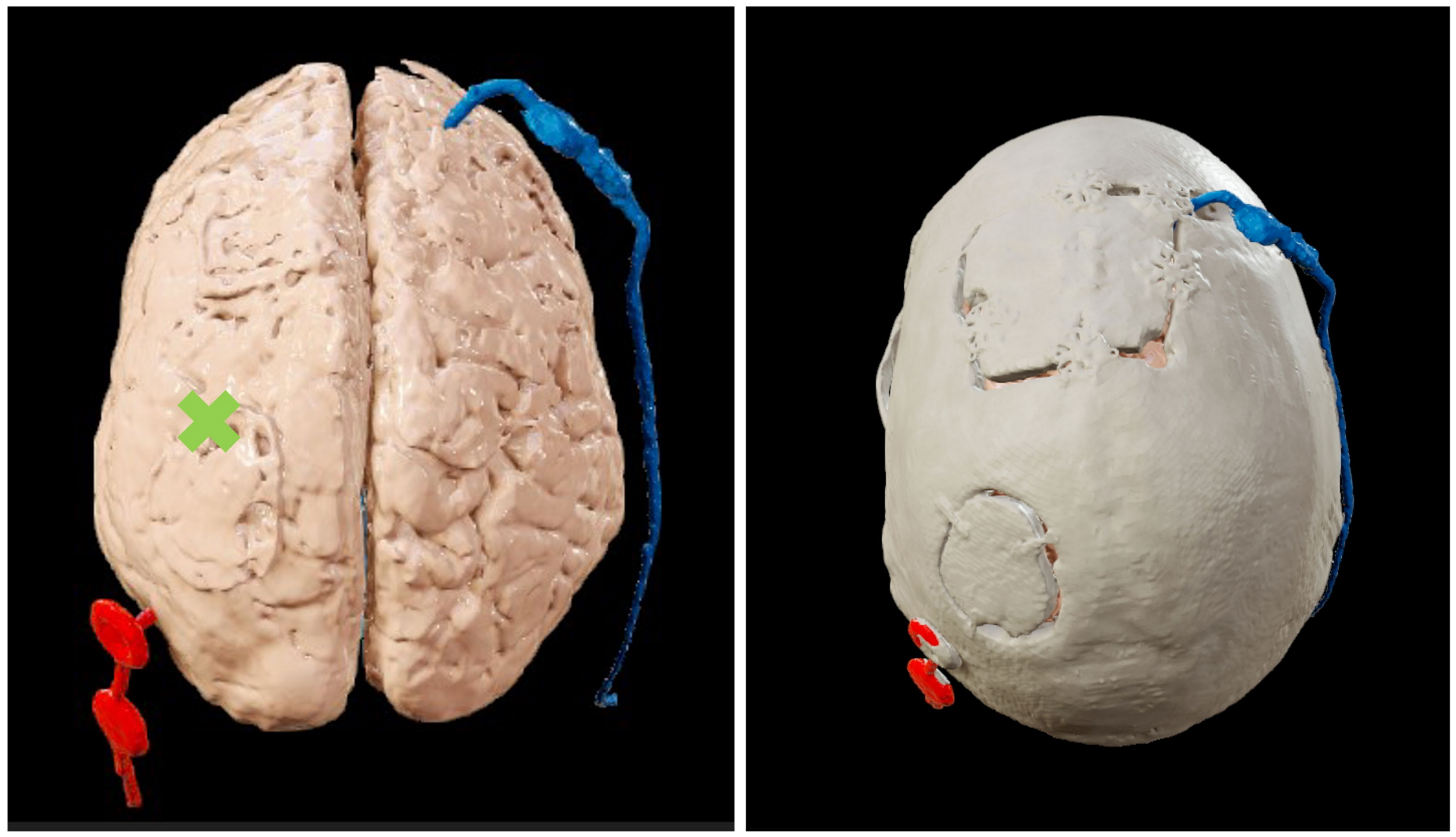
3D rendering demonstrating the location of the two ventriculoperitoneal shunts from patient 4. On the left, brain only rendering showing approximate location of the hand hotspot where the transcranial magnetic stimulation coil would be centered (green cross). On the right, skull rendering showing two craniotomy sites and at the edges of the craniotomies, metal skull fixation implants can be seen.

Patient 4 was a complex patient with two ventriculo-peritoneal shunts *in situ* to treat hydrocephalus secondary to two previous cranial operations (Figure 4). The right frontal valve was a Codman Certas™ programmable valve (Integra LifeSciences, Plainsboro, NJ, United States) and the left parietal was a Miethke proGAV 2.0® programmable valve (Aesculap, Tuttlingen, Germany). The Certas valve was set at 3 and the proGAV 2.0 was set at 5. Both valves use magnetic mechanisms to change the pressure settings. Figure 4 demonstrates the approximate location of where the TMS coil was centred over the left motor cortex relative to valves and the resection cavity. This patient’s valve settings were checked before the first and then after every rTMS session. No change in the valve settings was found throughout the whole rTMS treatment.

## Discussion

In this small preliminary study, we describe for the first time the use of ipsilesional high-frequency rTMS (IL-HFS) following surgical resection in four patients with intrinsic brain tumours. The intervention was well tolerated, with no serious adverse effects. Unusually, one patient reported transient paraesthesia of the unaffected foot, resolving within 24 h. Given the deep interhemispheric representation of the lower limb, high stimulation intensities are required to elicit motor responses, producing broader magnetic fields that may stimulate both hemispheres. The absence of focal motor symptoms and the persistence of paraesthesia beyond the stimulation period make it unlikely that this episode represented a seizure. The findings of this study provide preliminary evidence that IL-HFS can be safely administered in the early post-operative period. This study lays the groundwork for further clinical evaluation of IL-HFS in this population.

High-frequency stimulation in patients with brain tumours poses specific challenges that have prevented its exploration in the past. While the overall risk of rTMS-induced seizures is low when given within published guidelines —approximately 0.31 seizures per 10,000 sessions—the true risk in the post-operative tumour setting remains uncertain (20–22). In the early post-operative period, the peri-resection cortex will also be remodelling and therefore potentially hyperexcitable. Also, many patients will have pre-existing tumour-related epilepsy (23). Despite these theoretical risks, all four patients in our cohort tolerated IL-HFS without incident. This is particularly noteworthy despite the longer duration and increased number of pulses in the IL-HFS protocol. Caution is still due however given three patients were receiving prophylactic antiseizure medication and the rTMS was started relatively late (earliest at 17 days post-op) due to pragmatic constraints. Studies exploring rTMS in post-operative motor rehabilitation have tended to start rTMS much earlier (<72 h) (13, 14).

rTMS can also induce contractions of scalp or temporalis muscles, potentially exerting tension across healing wounds (15). The force of such contractions correlates with strength of the magnetic pulse and proximity of the muscle to the coil. This is of particular concern therefore with ipsilateral stimulation near recent craniotomy sites. by 1 week after wound closure, early collagen cross-linking gives surgical wounds ~3–5% of their final tensile strength rising to 20% at 3 weeks (24). Increase in tensile strength is sigmoidal and reaches its maximum around 6 weeks (25). Wound healing is compromised in revision cases or in patients with risk factors such as advanced age, steroid use, malnutrition, or smoking (26). In our cohort the earliest IL-HFS session commenced on day 17 and the latest was day 29 and no wound healing problems were found except in one patient. However, this patient was at high risk for wound breakdown even without rTMS as they had undergone two prior operations through the same incision. This case underscores the need for caution in such higher risk cases.

A further concern with coil proximity to the craniotomy site is metal implant heating due to Eddy currents or mechanical stress due to Lorentz forces (17). Modern plates and screws for closing craniotomies are primarily composed of titanium, a paramagnetic material that is MRI compatible (27). However, the magnetic fields induced by TMS are orders of magnitude larger than the gradient or transmit/receive fields used in MRI scanning (28). In a study assessing temperature change in two metal alloy vascular stents (nitinol and elgiloy) placed in gelled saline, neither 1 Hz nor 10 Hz rTMS induced a change of more than 1 degree Celsius (29). Theoretical finite element modelling indicates that used of rTMS with standard parameters near implanted ECOG grids or DBS electrodes does not generate significant force (~70mN) nor temperature changes (~0.02 Kelvin) (16). In an ex vivo experiment, no significant heating of titanium plates was found with 30-min of 1 Hz rTMS at 100% machine output, although significant heating was found with gold EEG electrodes (peak temperature ~42 degrees Celsius) (30). Other experiments have confirmed the significant heating of gold and silver based electrodes (31) with temperature changes likely proportional to duration, frequency, and intensity of stimulation (32). Gold and silver are diamagnetic and are therefore unlikely to experience significant Lorentz forces, however with an electrical conductivity an order of magnitude greater than that of titanium, they are susceptible to greater Eddy currents and heating (33). A study modelling the forces and temperature changes induced by various rTMS protocols on five common neurosurgical titanium-alloy implants demonstrated no significant mechanical forces but did indicate a temperature rise above the safety threshold in the titanium cranial mesh plate (34). The composition and size of the metallic implant is therefore crucial to consider. *In vivo*, the safety of low frequency 1 Hz rTMS at 100% of RMT near titanium skull plates in six patients receiving rTMS for epilepsy has been demonstrated (35). However, our cohort is the first to have high-frequency rTMS given close to the craniotomy site. None of our patients reported scalp pain or demonstrated wound changes suggestive of focal heating, supporting the view that this risk is negligible in clinical practice with titanium-based metallic implants.

The above discussion regarding Lorentz forces leads to the concern regarding the proximity of the coil to the programmable VP shunts in patient 4. Both implanted VP shunts in this patient utilise a magnetic mechanism to non-invasively change the pressure settings after surgical implantation. The settings on both shunts were checked regularly throughout the rTMS treatment course and at the end with no changes detected. This surprising finding is likely explained by the rapid decay over distance of magnetic fields. The Nexstim device can produce a magnetic field of up to 1.42 T at 6.5 mm from the coil bottom, induced by a biphasic pulse of duration 230 μs. At 20 mm depth, this maximal field has decayed to 0.66 T (36). Theoretically, the magnetic field generated by a circular or figure-8 coil follows an approximately 1/r^3^ dependence in free space, characteristic of a magnetic dipole. In practice, due to varying tissue conductivities and boundaries, both experimental mapping and modelling have demonstrated a more rapid, approximately exponential decay within the head, with characteristic length constants of 1–3 cm (37–39). Our study demonstrates that over the approximate distances shown in Figure 4, the effective magnetic field was too weak to induce any changes in VP shunt settings.

The rationale for exploring IL-HFS derives from advances in stroke rehabilitation. Conventional contralesional low-frequency stimulation (CL-LFS) aims to reduce excessive transcallosal inhibition from the unaffected hemisphere, thereby promoting recovery in the affected motor cortex (40, 41). However, an alternative strategy—direct excitatory stimulation of the ipsilesional hemisphere with high-frequency rTMS—has been shown to enhance activity in residual perilesional motor areas and to promote functional reorganisation, particularly within the viable penumbra (18, 42, 43). Recent studies further suggest that combining inhibitory and excitatory paradigms may offer additive benefit (44, 45). To date, only two randomised, sham-controlled trials have examined rTMS for post-operative motor rehabilitation in brain tumour patients, both employing CL-LFS protocols. One demonstrated clinical improvement (13), while the other found no significant difference from sham stimulation in the Fugyl-Meyer Scale, primary outcome of the study (14). No prior studies have investigated IL-HFS in this population. Given the accumulating evidence from stroke research supporting ipsilesional excitatory paradigms (18, 42, 43), evaluation of IL-HFS—alone or in combination with CL-LFS—is both timely and necessary. The present findings provide preliminary evidence that IL-HFS can be safely delivered in the early post-operative period, warranting further investigation in controlled trials.

## Limitations

This study has several limitations. Its retrospective design and absence of a control arm preclude differentiation between spontaneous recovery and stimulation effects. The small sample size limits statistical inference regarding motor or functional outcomes. Nonetheless, the principal objective of this study is sustained: to establish the feasibility and safety of IL-HFS in a post-operative brain tumour cohort. These results provide an essential foundation for future prospective studies with close observation further assessing the efficacy of IL-HFS stimulation.

## Conclusion

This study provides early evidence that ipsilesional high-frequency rTMS is feasible and well tolerated in the immediate post-operative period after resection of motor-eloquent brain tumours. It is important to maintain that this is a preliminary small-cohort study. Despite theoretical concerns regarding seizures, wound integrity, and hardware heating, no serious adverse effects were observed in this cohort. These findings justify cautious but proactive exploration of IL-HFS, alone or in combination with CL-LFS, in larger controlled studies to determine its efficacy in promoting functional motor recovery in this high-risk population.

## Data Availability

The original contributions presented in the study are included in the article/supplementary material, further inquiries can be directed to the corresponding author/s.
